# Munchausen Syndrome Disguised As Gossypiboma: An Interesting Case

**Published:** 2016-09-09

**Authors:** Andrea Little, Heather Curtis, Brian Kellogg, Michael Harrington

**Affiliations:** Department of Plastic Surgery, University of South Florida Morsani College of Medicine, Tampa; and H. Lee Moffitt Cancer Center, Tampa, Fla

**Keywords:** gossypiboma, surgical foreign body, Munchausen syndrome, factitious disorder, self-injurious behavior

## DESCRIPTION

**Figure 1:**
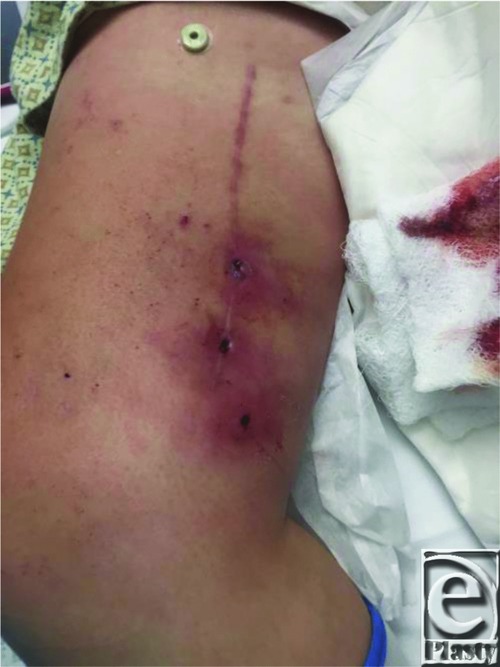
Recurrent cellulitis and draining sinus tracts of right medial thigh at previous gracilis flap donor site.

**Figure 2:**
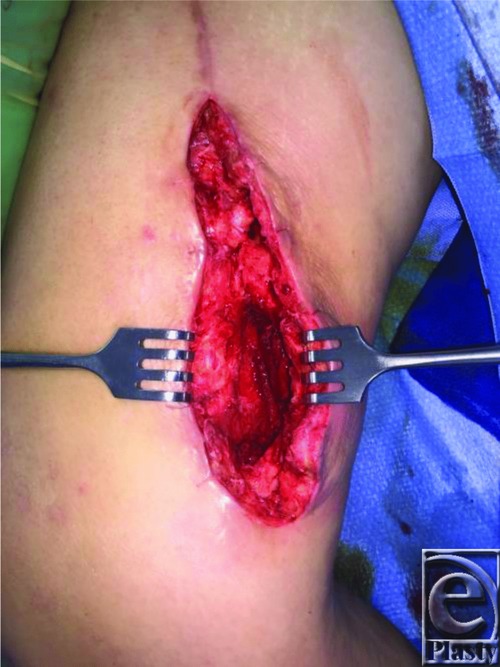
Intraoperative view of debridement of medial thigh wound.

**Figure 3:**
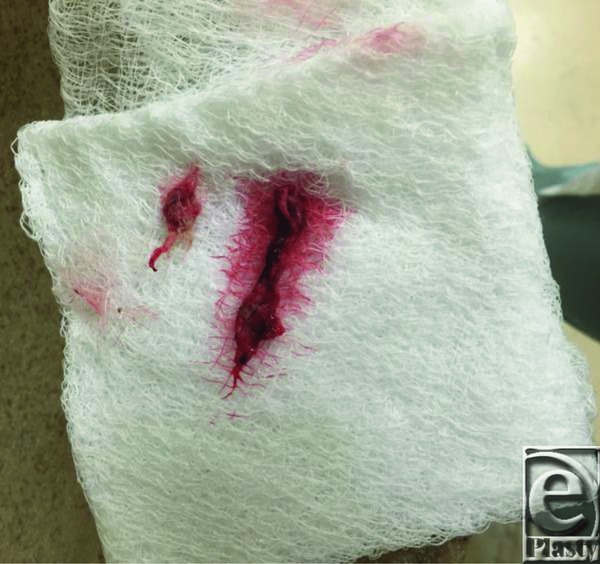
Intraoperative photograph of pieces of surgical gauze removed from medial thigh wound.

**Figure 4:**
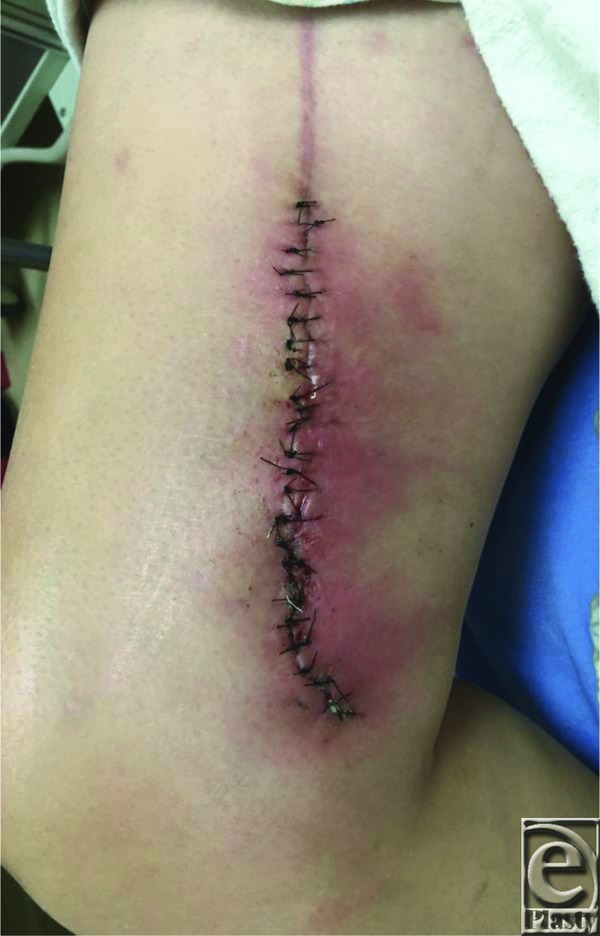
Incisional cellulitis of right medial thigh wound at emergency department presentation 2 weeks after initial irrigation and debridement with multilayer delayed primary closure.

A 45-year-old woman presented with recurrent cellulitis of a chronic right medial thigh wound at the site of a previous gracilis flap reconstruction (Fig 1). The donor site dehisced postoperatively and persisted despite numerous debridements, delayed primary closures, antibiotic regimens, and wound care. At the time of irrigation and debridement (Fig 2), several 2- to 3-cm fragments of gauze were removed from deep within the wound (Fig 3). Delayed primary closure was performed after subsequent debridements demonstrated no residual foreign body or infection. Two weeks later, the patient again presented with wound infection (Fig 4). Two fragments of gauze were encountered immediately under the surface of the incision during bedside irrigation & debridement (I&D). Subsequent management involved opening the wound, copious irrigation, removal of additional gauze, and vacuum assisted closure placement to prevent the patient from accessing her wound. A psychiatry consult was placed. Video observation showed no further abnormal behavior. Healing progressed without complication throughout the remainder of her inpatient course.

## QUESTIONS

1. **What are gossypiboma and Munchausen syndrome?**
2. **What signs/symptoms would cause consideration of Munchausen syndrome?**
3. **What are the medicolegal concerns for gossypiboma and Munchausen syndrome?**
4. **What are potential treatment options for self-injurious behavior?**

## DISCUSSION

Gossypiboma is a cottonoid mass surrounded by foreign body reaction, most commonly due to retained surgical sponge. The retained cotton matrix creates an inflammatory reaction by day 1 and progresses to granulomatous reaction and fibrosis after the first week.^1^ The majority of cases are postlaparotomy; however, cases have been reported in the thorax, thigh, and neck. Cautionary protocols such as sponge counts prior to closure of body cavities contribute to the rarity of this diagnosis.

Munchausen syndrome is a severe form of factitious disorder in which a person has recurrent, feigned, or self-inflicted illness to gain medical attention. It is a rare psychiatric diagnosis, particularly in postoperative patients.^2^ There is a higher incidence among women, and up to 50% are health care workers.^3^^,^^4^. It is unclear whether patients with Munchausen syndrome are conscious of their behavior. In contrast, malingering is another factitious disorder where patients consciously feign illness to obtain monetary gain in the form of litigation reward or workers' compensation.^3^

Signs that suggest Munchausen syndrome include unexplained course of disease, evidence of prior treatment, multiple health care providers, hospital and clinic visits at various locations, eagerness to undergo surgery, and an apparent desire to continue the sick role. Additional clues include unemployment or employment as a health care worker, history of assault, and other psychiatric diagnoses. Despite a thorough literature review, there are no published cases of Munchausen syndrome disguised as gossypiboma.

Medicolegal problems can arise in cases of gossypiboma, with patients claiming medical negligence because of the retained surgical sponge. In addition, the tendency for physicians to think within the framework of their specialty can delay a diagnosis of Munchausen syndrome. This can lead to litigation of the surgeon, consume facility resources, and expose the patient to additional invasive procedures. In this case, multiple providers failed to successfully heal the patient without infectious complications. Culture results showed primarily gram-negative organisms (*Klebsiella pneumoniae*, *Enterobacter cloacae*, *Escherichia coli*) atypical of soft-tissue infections. Since the wound was closed intraoperatively, no gauze should have been used to pack the wound. While most surgical sponges have a radiopaque marker and can be diagnosed on a plain radiograph,^1^ there was no radiographic evidence of foreign body in this case. In addition, the gauze removed was material not commonly used in operative settings. All sponge counts were correct at the end of her last 2 I&Ds, making it unlikely sizable pieces of gauze were missed despite 2 thorough washouts. It is also unlikely that residual gauze deep in the wound would have tracked to the surface in such a limited amount of time, as the wound was closed in multiple layers including muscle, muscle fascia, dermis, and epidermis. Relevant patient history included prior diagnoses of major depressive disorder and anxiety. Although she gave no history of abuse, recent life stressors included a new marriage approximately 1 month prior to her gracilis flap reconstruction. She did not initiate litigation or seek workers' compensation benefits.

Treatment of factitious disorders is challenging, especially in the context of comorbid psychiatric conditions. It is important to maintain a consistent team approach to reduce the patient's potential to split the team. Good rapport must be maintained to prevent self-discharge and representation of the patient elsewhere. Early involvement with psychiatrists prior to closure of the wounds is important to prevent reinjury and to assist in nonjudgmental confrontation of unusual behaviors.^5^ In addition to psychiatric consultation, consultation to forensic medicine specialists may help prevent possible legal repercussions.^2^
